# On Medical Reform

**Published:** 1813-09

**Authors:** 


THE
### Medical and Physical Journal.

3 OF VOL. XXX.] SEPTEMBER, 1813. [NO. 175.

### *To the Editors of the Medical and Physical Journal.*

GENTLEMEN,

I RELY on your wonted candor for the insertion of the following cursory remarks. The best view of the subject of Medical Reform, in my opinion, would be to advert to the utility that is derived from the profession in general, to every description of people, and to consider how it can be regulated to obtain the most possible good, not only to the profession, but to the people at large. Your correspondents, amongst many judicious and learned remarks, appear to me to have considered not so much the *bonum publicum*, as the interested opposition which exists between the colleges and the surgeon-apothecary. For that purpose the cobwebs have been brushed from ancient law-books, and enactments of less improved times are brought forward, to cripple the exertions, and destroy the interests of the surgeon-apothecary. According to these laws, the only authorised prescribers are the physicians, and "divers honest persons, as well men as women, (not surgeons and apothecaries,) whom God has endued with a knowledge of the nature, kind, and operation, of herbs, roots, and waters, to customable diseases, and who are allowed by enactment tempore Henry the Eighth, to use and minister the same, according to their cunning and knowledge." In modern language, these divers honest persons are water-casters, bone-setters, pretenders, and quacks, of the very lowest description, and generally believed by the vulgar to possess the occult art of sorcery and witchcraft; these have unrestrained and most extensive practice throughout the kingdom, except perhaps in surgery, within London, and seven miles round.

The surgeon-apothecary has been charged with overstepping the department of his profession. I do not believe that it has ever been in the most distant contemplation of the surgeon-apothecary to rival the physician, nor to charge in equal degree, but to legalize some inferior mode of remuneration for his attendance; for such is the nature of his department, that he commonly does and must attend prior, with, and subsequent to the physician. The surgeon-apothecary is never averse from the physician being called into consultation; it eases the burthen of responsibility, and adds in other respects largely to his interest. It is the legalized empirics empirics who refuse the co-operation of the physician; but, indeed, what physician would hold a consultation with them? Learning they despise, physicians and surgeons they are in the constant habit of ridiculing and nicknaming, and the confidence that the middle and lowest order of society have in these pretenders to the healing art, is exceedingly detrimental both to the public and the profession. No subject connected with medical economy calls more loudly for inquiry than this; these are the men to whom Salus Publica might more properly apply his epithets; it is they who are in possession of Pandora's box; they have opened it, and none can shut it but the powerful hand of an efficient act of parliament.

If the law of this realm, with regard to medical men, be as Censorinus would interpret it, then it follows that no man in this highly-favored country can be accommodated with medical advice, however dangerous or urgent his illness may be, unless he sends (if in a country place, and the patient able to bear the expenses of advice,) perhaps from ten to twenty miles for a physician; if not, however reluctant, he must, *per legem*, be content to submit his life to the ignorant herb-doctor. Does this call for no reform of the existing laws of the nation, with regard to the easiest and best manner of accommodating the people with medical assistance? It may be said these objections apply only to inconsiderable country situations, and that where population most abounds, the physician is at hand; but will the physician visit daily the poor man's garret or cellar, accompanied by his apothecary, (or his favorite druggist,) for about two shillings *per diem*? for this is the full amount of the apothecaries charges, medicines included: or could a sufficient number of physicians be found to attend such of the sick as refused the assistance of the herb-doctors, the other class of lawful practitioners? I am persuaded that there would not. If, then, the lives and healths of millions are to be regarded in preference to the monopoly of fees by any set of men; if it be desirable that the ignorant vulgar should have none but practitioners of acknowledged professional skill to make choice of; if it be right that the laborer is worthy of some lawful hire, and any weight is to be attached to reasonable argumentation, then the projected Medical Reform, in some prudent way, ought to be encouraged by every lover of his profession, and well-wisher of the public. I am, Gentlemen,

Your most obedient Servant,

ANTIEMPIRICUS.

*Rochdale, July* 9, 1813.

